# Art27 Interacts with GATA4, FOG2 and NKX2.5 and Is a Novel Co-Repressor of Cardiac Genes

**DOI:** 10.1371/journal.pone.0095253

**Published:** 2014-04-17

**Authors:** Daniel R. Carter, Andrew D. Buckle, Kumiko Tanaka, Jose Perdomo, Beng H. Chong

**Affiliations:** 1 Centre for Vascular Research, Department of Medicine, St. George Clinical School, University of New South Wales, Sydney, New South Wales, Australia; 2 Haematology Department, St George and Sutherland Hospitals, University of New South Wales, Sydney, New South Wales, Australia; Maastricht University Faculty of Health, Medicine, and Life Sciences, Netherlands

## Abstract

Transcription factors play a crucial role in regulation of cardiac biology. FOG-2 is indispensable in this setting, predominantly functioning through a physical interaction with GATA-4. This study aimed to identify novel co-regulators of FOG-2 to further elaborate on its inhibitory activity on GATA-4. The Art27 transcription factor was identified by a yeast-2-hybrid library screen to be a novel FOG-2 protein partner. Characterisation revealed that Art27 is co-expressed with FOG-2 and GATA-4 throughout cardiac myocyte differentiation and in multiple structures of the adult heart. Art27 physically interacts with GATA-4, FOG-2 and other cardiac transcription factors and by this means, down-regulates their activity on cardiac specific promoters α-myosin heavy chain, atrial natriuretic peptide and B-type natriuretic peptide. Regulation of endogenous cardiac genes by Art27 was shown using microarray analysis of P19CL6-Mlc2v-GFP cardiomyocytes. Together these results suggest that Art27 is a novel transcription factor that is involved in downregulation of cardiac specific genes by physically interacting and inhibiting the activity of crucial transcriptions factors involved in cardiac biology.

## Introduction

Cardiomyocytes are maintained by intricate molecular regulatory programs that involve a multitude of transcription factors. Heart development utilises conserved transcription factor families such as GATA, Nk2, HAND, MEF2 and TBX as the central hub of regulation [1]. Interestingly reactivation of some of these developmental regulators such as GATA factors is crucial to promoting the cardiac hypertrophy disease state suggesting functional activity is maintained into adulthood [2].

GATA-4, a well-known enhancer of cardiac development [3] has an indispensable functional interaction with FOG-2. FOG-2 and GATA-4 are co-expressed in both the developing and adult heart [4] and FOG-2 regulates GATA-4 transcriptional activity on cardiac specific genes atrial natriuretic peptide (ANP), b-type natriuretic peptide (BNP) and alpha myosin heavy chain (αMHC) [4], [5]. FOG-2 deficient murine embryos have severe cardiac malformations resulting in embryonic lethality [6], [7] and this phenotype is recapitulated to a large extent in transgenic GATA-4 embryos that have a knock-in mutation that prevents a GATA-4/FOG-2 interaction [8]. Similarly, FOG-2 polymorphisms are associated with the congenital heart disease Tetralogy of Fallot revealing conserved FOG-2 function in human heart development [9]. In addition to their role in development, GATA-4 and FOG-2 have functional roles in regulation of the adult heart, both shown to participate in the regulation of cardiac hypertrophy [2], [10], [11].

Given the importance of FOG-2 in cardiomyocyte biology as a GATA-4 cofactor, we hypothesised that FOG-2 may bridge other novel transcription factors into the cardiac regulatory network as a protein cofactor. Thus we aimed to identify novel protein interaction partners for FOG-2. Subsequently, a novel cardiac transcription factor called Art27 was identified.

Art27 (also known as UXT) is a 157 amino acid protein with a broad expression profile, with particularly strong expression in cardiac tissue [12], [13]. It has been shown to be nuclear localised [12], [14] and has a high intrinsic binding affinity to other proteins such as the androgen receptor, EVI1, NFκB and others [12], [14], [15], [16], [17], [18]. Art27 acts as a transcriptional co-regulator, targeting genes important in regulation of the cell cycle, cell proliferation and inflammation [14], [17], [19], [20].

In this study we showed that a yeast-2-hybrid library screen with FOG-2 identified an Art27/FOG-2 physical interaction and that by this mechanism Art27 enhances the FOG-2-mediated transcriptional repression of GATA-4. Furthermore, Art27 was identified to physically interact and downregulate GATA-4 activity on cardiac specific promoters independently of FOG-2 and similarly repressed the activity of cardiac transcription factor Nkx2.5, as well as other GATA factors, GATA-6 and GATA-1. Moreover, knockdown of Art27 in P19CL6-Mlc2v-GFP cardiomyocytes resulted in upregulation of key cardiac regulatory transcripts, Sfrp4, Wnt11, histamine receptor 2 and endothelin 3 as assessed by microarray analysis. Thus we propose that Art27 is a novel regulator of cardiac genes that acts by physically interacting with cardiac transcription factors.

## Results

### A FOG-2 Yeast-2-hybrid Library Screen Reveals a Physical Interaction with Art27

To identify novel FOG-2 binding partners, a yeast-2-hybrid screen was undertaken. The library chosen for screening was based on clones derived from a human heart cDNA library. The library screen was undertaken using a C-terminal expression construct of FOG-2 (amino acids 856–1156). This region was chosen as it failed to auto-activate yeast promoters. Bait and prey constructs were transformed into the AH109 yeast strain and plated on SD-media agar lacking essential growth nutrients that selected for novel physical interactions. Subsequently, 54 candidate clones were identified. The novel FOG-2 interactions identified are summarised in **Table 1**.

**Table 1:** FOG-2 protein binding partners from yeast-2-hybrid screen.

CLONE	PUTATIVE IDENTITY	NCBI ACCESSION #
1	ART-27	NM_153477
2	ART-27	NM_153477
3	Collagen α1 polypeptide	NM_000088
4	Collagen α1 polypeptide	NM_000088
5	No significant homology found	
6	KIAA0657	XM_051017
7	Phytanoyl-CoA hydrolase	NM_006214
8	Phytanoyl-CoA hydrolase	NM_006214
9	Fibronectin-1 (FN-1)	NM_212482
10	Fibronectin-1 (FN-1)	NM_212482
11	Human fibronectin 1 splice variant	NM_002026
12	Four and a half LIM protein 2 (FHL2)	NM_001450
13	Four and a half LIM protein 2 (FHL2)	NM_001450
14	α-cardiac actin	NM-006214
15	KIAA1662	AB051449
16	Titin (TTN)	BC070170
17	Collagen Type 2 (COLIA2)	NM_000089.3
18	Nebulette (NEBL)	NM-006393
19	Four and a half LIM protein 2 (FHL2)	NM_001450
20	Filamin-C (FLNC)	NM_001458
21	Four and a half LIM protein 2	NM_001450
22	Ubiquitin conjugating enzyme (UBC9)	NM_194261
23	Fibronectin-1 (FN-1)	NM_212482
24	Filamin-C (FLNC)	NM-001458
25	No significant homology found	
26	Four and a half LIM protein 2 (FHL2)	NM_001450
27	Phytanoyl-CoA hydrolase	NM_006214
28	Four and a half LIM protein 2 (FHL2)	NM_001450
29	Four and a half LIM protein 2 (FHL2)	NM_001450
30	Four and a half LIM protein 2 (FHL2)	NM_001450
31	Fibronectin 1 (FN-1)	NM_212482
32	Fibronectin 1 (FN-1)	NM_212482
33	Four and a half LIM protein 2 (FHL2)	NM_001450
34	No significant Homology Found	
35	No Significant Homology Found	
36	Four and a half LIM protein 2 (FHL2)	NM_001450
37	Nebulette (NEBL)	NM-006393
38	Four and a half LIM protein 2 (FHL2)	NM_001450
39	Fibronectin 1 (FN-1)	NM_212482
40	No significant homology found	
41	Collagen type 1	NM_000088
42	Disulphide Isomerase related (P5)	D49489
43	ART-27	NM_153477
44	Four and a half LIM protein 2 (FHL2)	NM_001450
45	Four and a half LIM protein 2 (FHL2)	NM_001450
46	Collagen type 1	NM_000088
47	Elastin Myofibril Interfacer Protein	BC009947
48	Elastin Myofibril Interfacer Protein	BC009947
49	KIAA1426	AB037847
50	Four and a half LIM protein 2 (FHL2)	NM_001450
51	Four and a half LIM protein 2 (FHL2)	NM_001450
52	No significant homology	
53	Nebulette (NEBL)	NM-006393
54	Serine Protease Inhibitor (SERP1NF1)	NM_002615

Analysis of the positive interacting sequences revealed a novel physical interaction with transcriptionally active proteins four and a half lim protein 2 (FHL2) and Art27. Subsequent functional characterization studies focused on the consequence of the FOG-2/Art27 physical interaction. The FOG-2/FHL2 interaction is the subject of a separate study.

**Figure 1:**
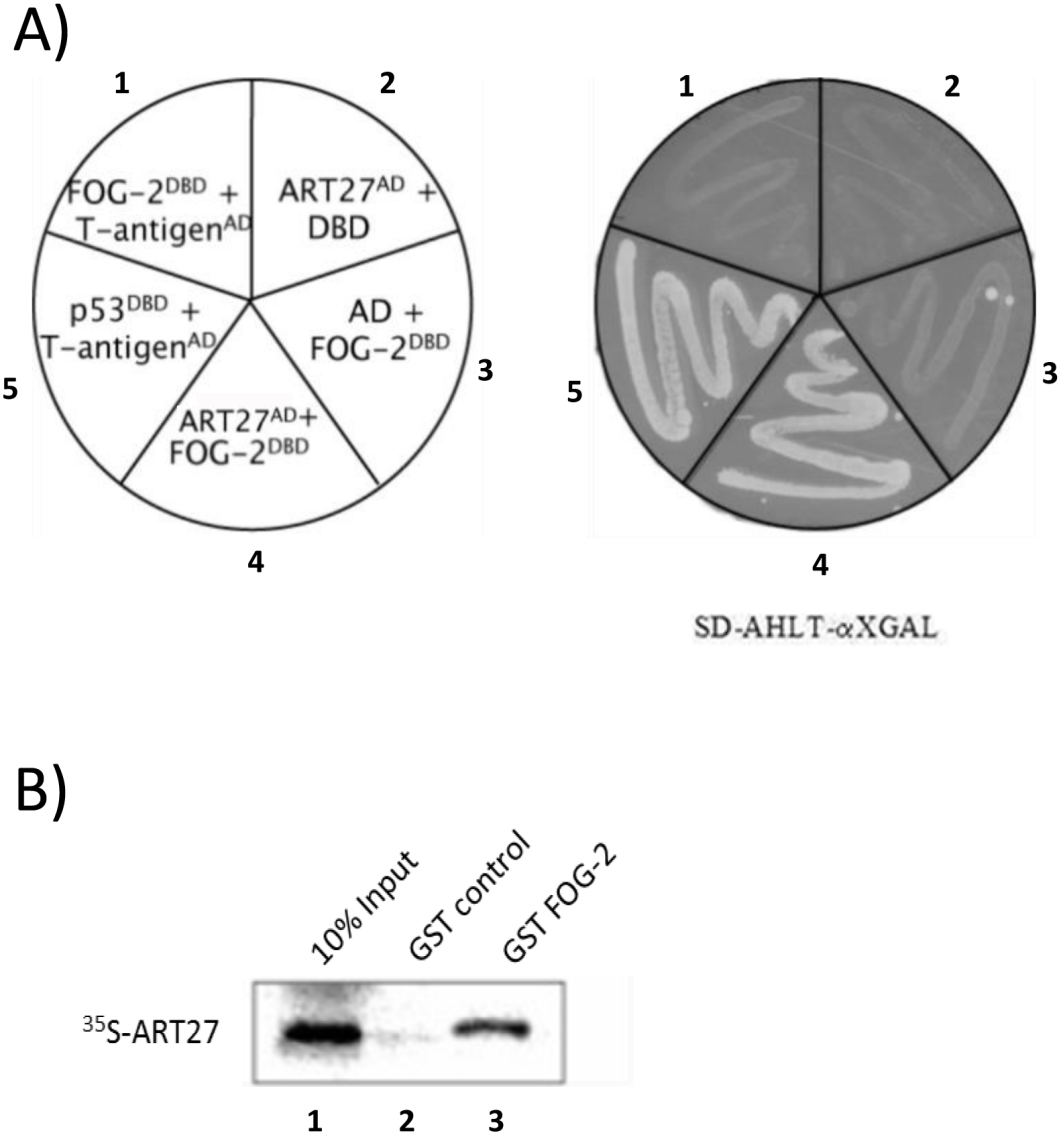
FOG-2 physically interacts with Art27. (**A**) AH109 yeast were transformed with the respective bait and prey constructs and plated on synthetic dropout media lacking adenine, histidine, leucine and tryptophan and tested for X-GAL positive yeast growth. FOG-2 (amino acids 856**–**1156) failed to physically interact T-antigen (segment 1- negative control), Art27 and FOG-2 (856**–**1156) failed to autoactivate yeast growth (segment 2 and 3 respectively), Art27 and FOG-2 (856**–**1156) physically interact and promote yeast growth (segment 4) and as expected the physical interaction between p53 and T-antigen promoted yeast growth (segment 5 – positive control). (**B**) *In vitro* translated and ^35^S radiolabeled Art27 protein was incubated with full length FOG-2/GST fusion protein or GST that was immobilised on glutathione sepharose beads. After extensive washing the proteins were resolved by electrophoresis and detected using a phosphorimager. ^35^S labelled Art27 was retained only by the FOG-2/GST fusion protein (lane 3) indicating that they physically interact.

Prior to further functional analysis, the validity of the FOG-2/Art27 interaction was confirmed. Yeast-2-hybrid was repeated with rescued full-length Art27 as prey and FOG-2 (856–1156) as bait. First, we confirmed that neither clone was capable of auto-activation (**Figure 1A**). Upon co-transfection of Art27 and FOG-2 (856–1156), however, strong yeast growth was observed, indicating the physical association between these molecules **(** **Figure 1A** **)**. Similarly, a physical interaction was confirmed *in vitro* between full-length FOG-2 and Art27 by GST-pulldown assay (**Figure 1B**).

### Art27 Shows Characteristics of a Cardiac Transcription Factor

Art27 has the fundamental hallmarks of a transcriptional regulator. For example Art27 is found in the nucleus [12], [14], is able to regulate specific promoters [12], [14] and has a high binding affinity for several other factors [12], [14], [15], [16], [17]. Additionally, the high level of cardiac tissue expression associated with Art27 and its long evolutionary heritage [12], [13] render Art27 a possible candidate for function within the cardiac regulatory network.

**Figure 2:**
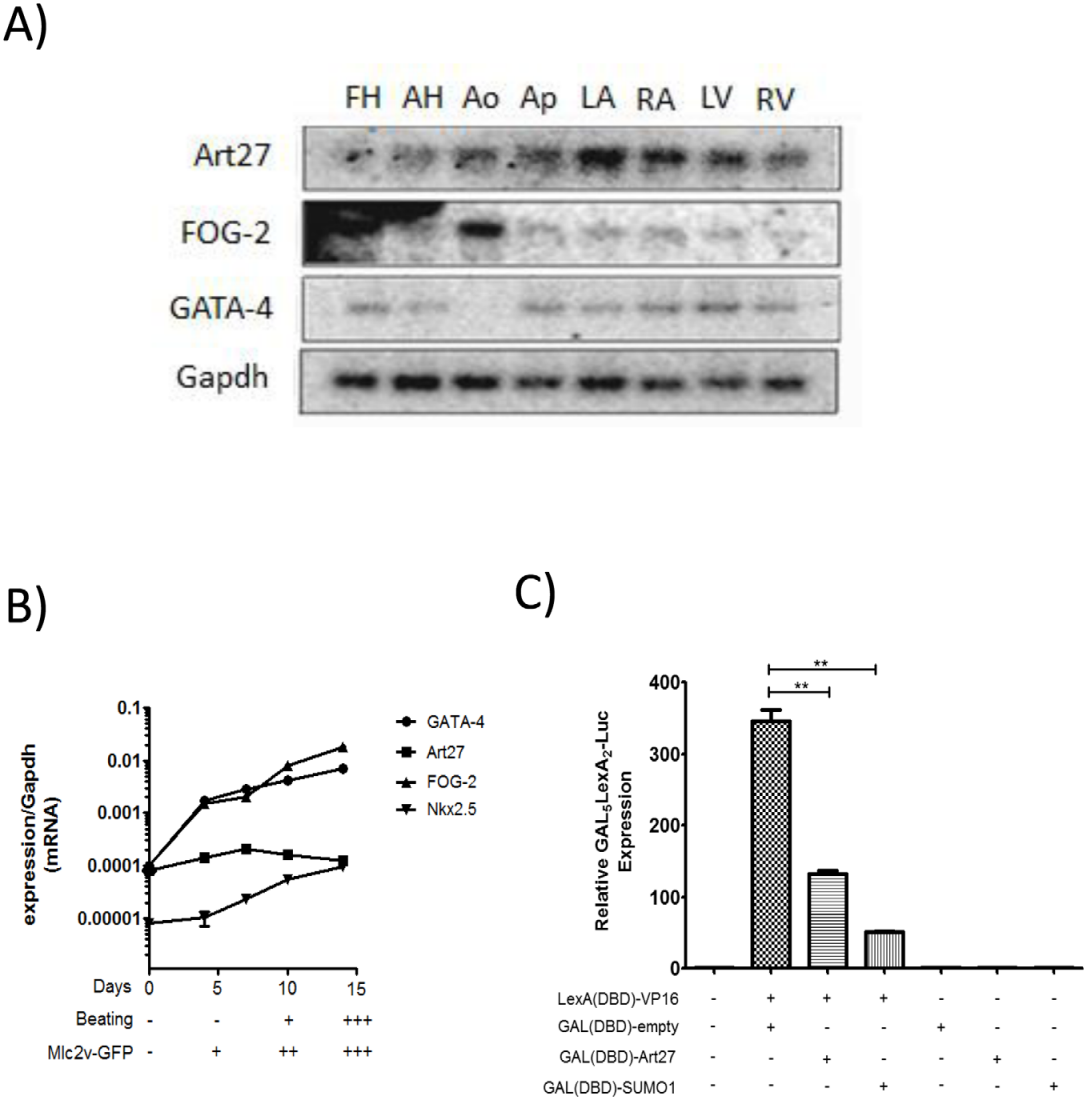
Art27 is a potential transcriptional regulator in cardiac developmental and adult tissues. (**A**) Northern blot on RNA derived from eight different heart tissues was probed for expression of GATA-4, FOG-2 and Art27. The membrane was stripped and re-blotted with the appropriate label. The housekeeping gene Gapdh was used as a loading control. Differential binding and/or absence of binding is indicative of probe specificity. (**B**) P19CL6 embryonal carcinoma cells with stably incorporated GFP under the control of the cardiac Mlc2v promoter [26] were induced to undergo cardiomyocyte differentiation with 1% DMSO. At 5-day increments, mRNA was isolated to assess relative expression of cardiac transcription factors Art27, GATA-4, FOG-2 and Nkx2.5 compared to housekeeping gene Gapdh. These time points correspond to various stages of cardiomyocyte differentiation as determined by systematic scoring of GFP fluorescence and beating intensity. (**C**) 293a cells were transfected with the various expression plasmids as indicated and GAL_5_LEXA_2_-Luc reporter activity was assessed. GAL(DBD)-Art27 significantly diminishes reporter activity compared to GAL(DBD)-empty expression plasmid alone showing inherent transcriptional repression activity. GAL(DBD)-SUMO-1 was used as positive control and was observed to repress the activity of the reporter as expected [49] **p<0.01. FH, foetal heart, AH, adult heart, Ao, aorta, Ap, apex, LA, left atrium, RA, right atrium, LV, left ventricle, RV, right ventricle.

In order to determine if Art27 has the characteristics of a cardiac transcription factor, a number of characterization assays were conducted. To assess expression of Art27 in specific human cardiac structures a multi-tissue northern blot was conducted for Art27 and FOG-2, as well as the FOG-2 cofactor GATA-4 (**Figure 2A**). Of the eight tissues analysed (foetal heart, adult heart, aorta, apex, left atrium, right atrium, left ventricle and right ventricle), Art27 is expressed in all tissues with particularly high expression in the left and right atrium and it is co-expressed with FOG-2 and GATA-4 in all tissues except the aorta, where no GATA-4 mRNA is detected (**Figure 2A**). To analyse the expression of Art27 throughout developing cardiac tissues we utilised the P19CL6 cardiomyocyte differentiation system [21]. mRNA expression for Art27, FOG-2, Nkx2.5 and GATA-4 was quantified at various time-points relative to housekeeping gene Gapdh using conditions consistent with previously published methodology [22], [23]. GATA-4, FOG-2, Nkx2.5 and Art27 were identified to be co-expressed at all time-points assessed (**Figure 2B**). Consistent with previous reports, GATA-4 and Nkx2.5 were upregulated through cardiomyocyte differentiation [23], while Art27 demonstrated consistent expression throughout differentiation. These findings show that Art27, GATA-4, Nkx2.5 and FOG-2 have overlapping expression in developing and adult cardiac tissues, indicating the possibility of functional interactions *in vivo*.

To evaluate Art27’s intrinsic transcriptional activity, a GAL4-DNA binding domain-Art27 plasmid (GAL(DBD)-Art27) was assessed in a transactivation assay using GAL4 responsive luciferase reporters. When GAL(DBD)-Art27 was transfected with the GAL_5_-luciferase reporter alone, no detectable change in luciferase activity was detected for either construct (data not shown). However when GAL(DBD)-Art27, LexA(DBD)-VP16 and the LexA_2_GAL_5_-luciferase reporter were co-transfected, GAL(DBD)-Art27 dramatically down-regulated the LexA(DBD)-VP16 activated reporter (p<0.05) (**Figure 2C**). This demonstrates that Art27 has intrinsic transcriptional repression function.

These experiments show that Art27 is co-expressed with cardiac transcription factors in multiple structures of the adult heart and throughout P19CL6 cardiomyocyte differentiation. These results, together with the observation that Art27 possesses intrinsic transcriptional repressor activity support the proposition that Art27 is a cardiac transcriptional co-factor and that Art27 may regulate cardiac genes with FOG-2 and GATA-4.

### Art27 Downregulates Cardiac Genes with FOG-2 and GATA-4

**Figure 3:**
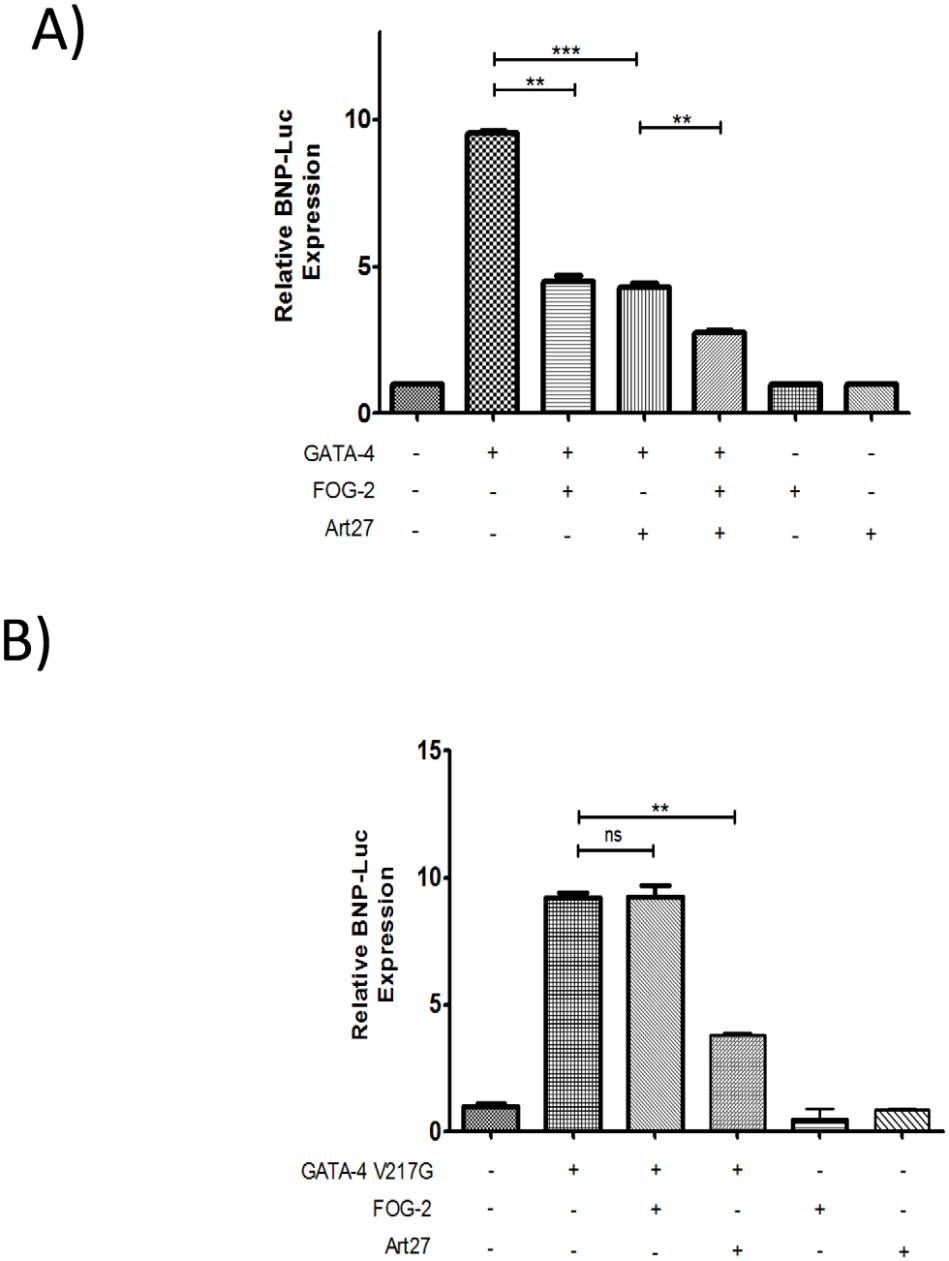
Art27 transcriptionally represses GATA-4 independently of FOG-2. 293a cells were transfected with the various expression plasmids as indicated and relative transactivation was conducted using luciferase reporter assays. (**A**) Art27 and FOG-2 significantly diminish GATA-4 transactivation of the Bone Natriuretic Peptide luciferase (BNP-Luc) reporter independently and also additively when cotransfected. (**B**) GATA-4 V217G transactivation of the BNP-Luc reporter is significantly repressed by Art27 but not FOG-2.

FOG-2 primarily functions through an interaction with GATA-4 and in luciferase assays, represses the GATA-4 activation of a number of cardiac promoters including b-type natriuretic peptide (BNP) [4], [5], [24]. To assess Art27 function in the presence of cardiac promoters, transfection of expression plasmids for Art27, FOG-2 and GATA-4 was undertaken with the BNP-luciferase reporter as a representative cardiac promoter (**Figure 3A**). As seen in previous studies, GATA-4 stimulates BNP transcription and the addition of FOG-2 diminishes this transactivation [4], [5], [24]. Consistent with Art27’s repression activity shown in Figure 2C, co-expression of Art27 with GATA-4 led to potent inhibition of GATA-4-mediated activation. Moreover, expression of Art27 together with FOG-2 and GATA-4 significantly enhanced transcriptional repression, indicating that Art27 also acts as a transcriptional co-repressor (**Figure 3A**).

Figure 3A suggests that Art27 may have the capacity to directly repress GATA-4 activity. To actively investigate the means of Art27 repression of GATA-4, a GATA-4 construct that contains the V217G mutation was used. Mutation of this valine residue abrogates the FOG-2 interaction capacity of GATA-4 [8]. This construct is useful as it still maintains DNA binding and transcriptional activity but loses its capacity to be repressed by FOG-2 [8]. By this means it is possible to investigate whether Art27 repression of GATA-4 is FOG-2 dependent. When GATA-4 V217G and BNP-luciferase were co-transfected, the increase in luciferase activity was indistinguishable from that of wildtype GATA-4 (**Figure 3A and 3B**). As expected for this mutant, co-expression of FOG-2 did not repress the activity of GATA-4 V217G [8] (**Figure 3B**). However when Art27 expression plasmid was transfected, GATA-4 V217G induced luciferase activity was significantly diminished (**Figure 3B**). Thus, Art27 can repress GATA-4 activity independently of FOG-2.

### Art27 Physically Interacts with Cardiac Transcription Factors

**Figure 4:**
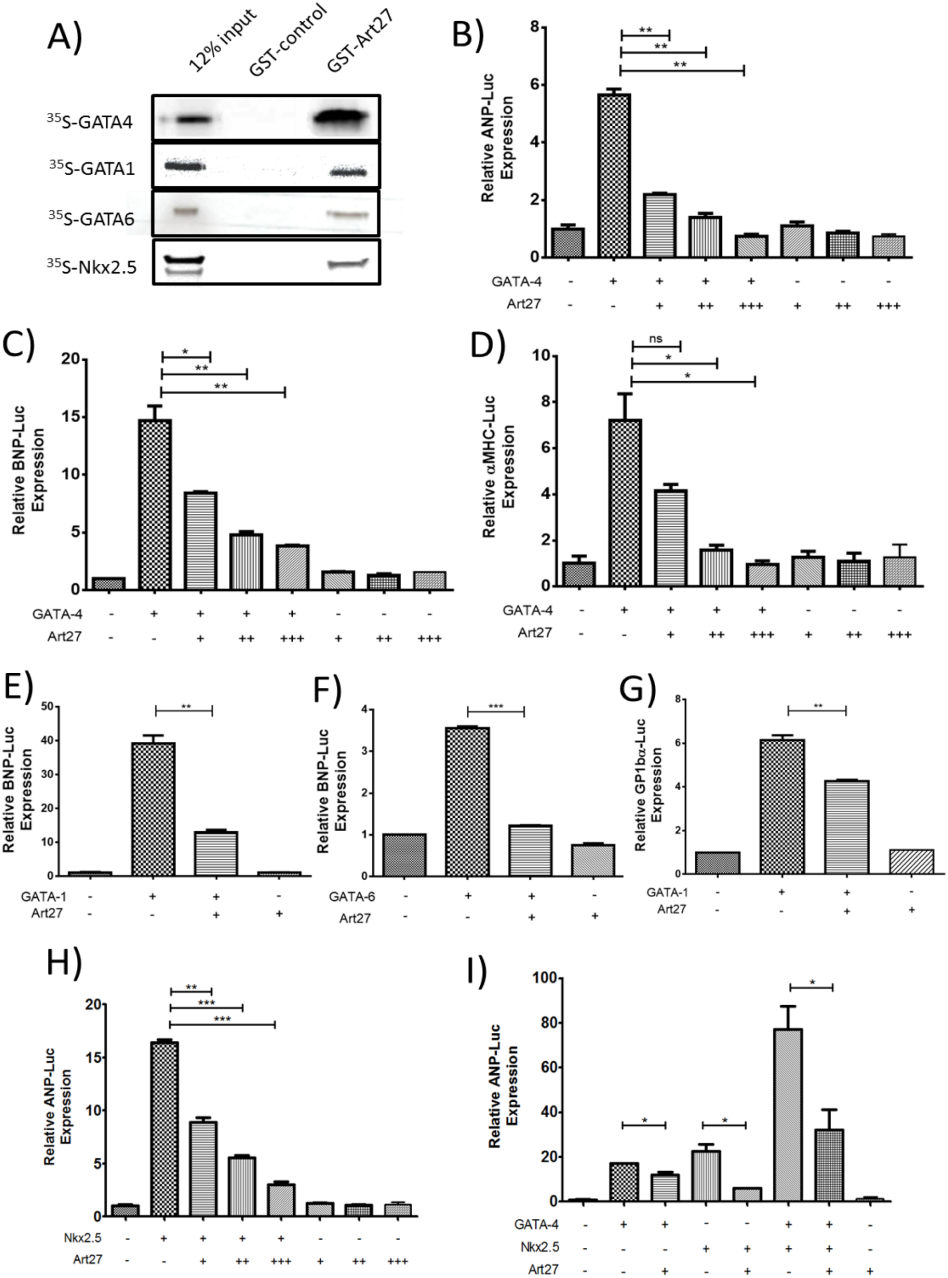
Art27 physically interacts and transcriptionally represses cardiac transcription factors. (**A**) *In vitro* translated and 35S radiolabeled GATA-4, GATA-1, GATA-6 or Nkx2.5 was incubated with full length Art27/GST fusion protein (GST-Art27) or GST-only (GST-control) that was immobilised on glutathione sepharose beads. After extensive washing and electrophoresis, phosphorimaging identified the presence of all factors tested with GST-Art27 but not with GST-control suggesting that they physically interact. (**B–I**) 293a cells were transfected with Art27 and various other cardiac transcription factors as indicated and luciferase transactivation assays were conducted with a variety of reporters. Art27 represses GATA-4 in a dose dependent manner on atrial natriuretic peptide luciferase (ANP-Luc) reporter (B), BNP-Luc (C), and alpha myosin heavy chain luciferase (αMHC) reporter (D). Art27 represses GATA-1 and GATA-6 on the BNP-luc reporter (E and F respectively) and GATA-1 on the glycoprotein 1b alpha luciferase (GP1bα-Luc) reporter (G). Art27 represses Nkx2.5 in a dose dependent manner on the ANP-Luc reporter (H). Art27 represses a GATA-4/Nkx2.5 synergistically transactivated ANP-Luc reporter (I). *, p<0.05, **, p<0.01, ***, p<0.001.

Given that Art27 regulates GATA-4 through a mechanism independent of FOG-2 interaction and Art27 has been identified as a frequent protein cofactor in other studies [12], [14], [15], [16], [17], [18], we used GST pulldown assays to investigate whether Art27 physically interacts with cardiac transcription factors GATA-4, Nkx2.5 and GATA-6, as well as the haematopoietic GATA transcription factor GATA-1. In all assays, a physical association was identified between Art27 and the tested constructs (**Figure 4A**).

To more specifically detail the Art27/GATA-4 interaction, GST-pulldown assays revealed that Art27 interacted with the GATA-4 region of amino acids 217–440 but not amino acids 1–216 (**Figure S1A**) and yeast-2-hybrid of the GATA-4 N-terminal and C-terminal zinc fingers revealed that Art27 can bind either of these regions (**Figure S1B**), suggesting that the association between Art27 and GATA-4 involves multiple contact residues.

### Art27 Downregulates the Activity of Cardiac Transcription Factors

For functional analysis of the identified Art27 physical interactions, luciferase transactivation assays were utilised. Using a variety of promoters we found that Art27 was able to repress the activity of all interacting partners. Art27 was shown to repress GATA-4 activity in a dose dependent manner on known cardiac promoters such as atrial natriuretic peptide (ANP), BNP and alpha myosin heavy chain (αMHC) luciferase reporters (**Figure 4B, C and D** **, respectively**). Similarly, other GATA factors, GATA-1 and GATA-6 are repressed on the BNP promoter (**Figure 4E and 4F** **, respectively**) and GATA-1 is repressed on the megakaryocytic specific glycoprotein 1bα (GP1bα) promoter (**Figure 4G**). Consistent with Art27 acting as a repressive cofactor, Nkx2.5 is also repressed by Art27 in a dose dependent manner on the ANP promoter (**Figure 4H**). Collectively, these data demonstrate the transcriptional repression capacity of Art27 on a variety of promoters.

In addition to investigating Art27 with the above transcription factors individually, Art27 transcriptional repression was assessed when co-transfected with both GATA-4 and Nkx2.5. Together GATA-4 and Nkx2.5 act synergistically to amplify ANP transcription by forming protein-protein stimulatory complexes [25]. When Art27 was added to the GATA-4/Nkx2.5 synergy complex, luciferase activity was significantly diminished (**Figure 4I**), again demonstrating Art27 as a novel cardiac transcriptional repressor.

To confirm that Art27 mediated downregulation of the cardiac transcription factors was not an artefact of transfection of exogenous expression plasmids, GATA and Nkx2.5 expression was analysed whilst co-transfected with Art27. Art27 cotransfection had no repressive effects on relative transgene expression for either GATA-4 or Nkx2.5 (**Figure S2A and S2B, respectively**) suggesting that Art27’s repressive activity takes place through physical interaction that impairs the transactivation function of cardiac transcription factors.

### Art27 Knockdown Increases Expression of Endogenous Cardiac Genes

**Figure 5:**
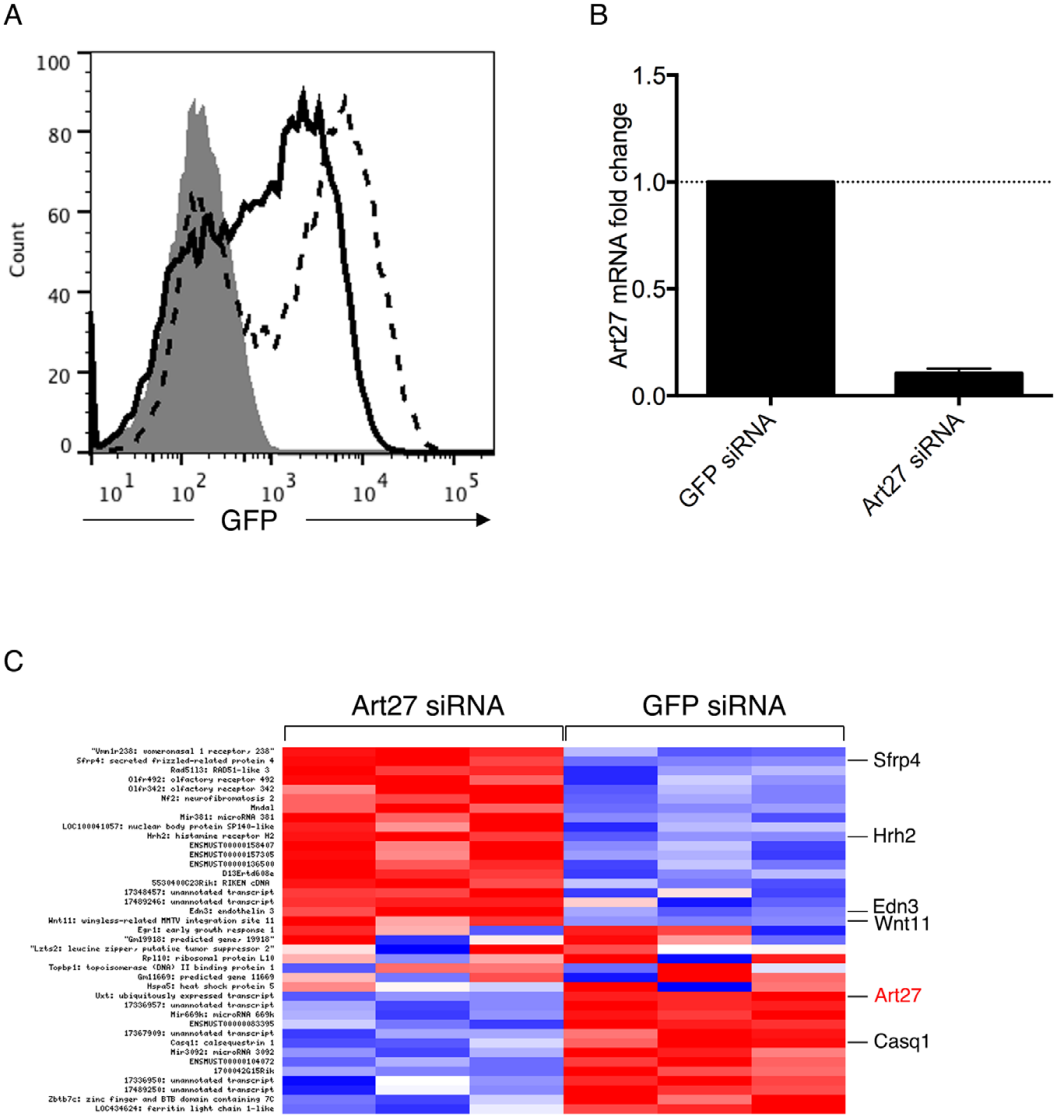
Art27 knockdown de-represses endogenous cardiac genes. (**A**) Differentiation of P19CL6-Mlc2v-GFP cells was monitored by flow cytometry. The solid histogram represents GFP fluorescence of undifferentiated cells (Day 0). The solid and dotted lines show GFP fluorescence on differentiation Days 7 and 11, respectively. (**B**) Art27 knockdown relative to control. The level of Art27 mRNA was measured by qPCR in differentiated P19CL6-Mlc2v-GFP cells (Day 7) nucleofected with control siRNA (GFP siRNA) or specific Art27 siRNA. Nucleofection with Art27 siRNA resulted in a 10-fold reduction of Art27 mRNA. (**C**) Heatmap showing the relative expression of transcripts that were significantly upregulated (red) or downregulated (blue) in differentiated P19CL6-Mlc2v-GFP cells (Day 7) nucleofected with Art27 siRNA. Relevant cardiac transcripts are indicated.

We used siRNA-mediated down-regulation to evaluate the effect of the absence of Art27 in P19CL6-Mlc2v cells differentiated for 7 days in the presence of 1% DMSO. **Figure 5A** shows GFP expression as an indication of P19CL6-Mlc2v-GFP cell differentiation into cardiomyocytes [26]. Treatment of differentiated cells with Mission esiRNA against Art27 resulted in marked down-regulation (10-fold) of Art27 message relative to control esiRNA (against GFP) (**Figure 5B**). mRNA was harvested 36 h post-nucleofection and used for microarray analysis using Affymetrix Mouse Gene Arrays 2.0ST. The experiments were performed in triplicate. **Figure 5C** shows the heatmap of transcripts showing significant upregulation in Art27 siRNA cells. Of the genes upregulated upon Art27 knockdown, secreted frizzle-related protein 4 (Sfrp4), histamine receptor 2, endothelin 3 and Wnt11 are crucial factors involved in cardiac differentiation and function [27], [28], [29], [30]. Non-canonical Wnt11 signalling is required for cardiogenesis in *Xenopus* and also triggers cardiogenesis in mouse P19 cells [30]. Moreover, Sfrp4 is the most upregulated gene in Art27 siRNA knockdown cardiomyocytes (P = 0.0001). Sfrp is a central regulator of Wnt11 signalling [31] and its high expression, together with Wnt11, suggests that Art27 is a repressor of cardiac genes and is likely to be involved in the maintenance of required expression levels.

## Discussion

This study identified Art27 as a novel transcriptional co-regulator of cardiac specific genes. Art27 was seen as a recurrent transcriptional cofactor to important cardiac regulators including members of the GATA family, FOG-2 and Nkx2.5. The mechanism for Art27 transcriptional activity was by protein-protein interaction where transcription factors recruit Art27 for repression of cardiac (ANP, BNP and αMHC) promoters. These particular genes are well known to have roles in many facets of cardiac physiology so Art27 may potentially be an important cardiac regulator. Additionally, this study identified that Art27 is expressed in the foetal heart and all major structures of the adult heart, as well as throughout P19CL6 cardiomyocyte differentiation suggesting possible functional activity *in vivo*. Moreover, in P19CL6-Mlc2v-GFP cardiomyocytes, Art27 was shown to modify the expression of cardiac differentiation genes including Sfrp4, histamine receptor 2, endothelin 3 and Wnt11.

Art27 has a high capacity to physically interact with other proteins. In this study it was found to physically associate with FOG-2, GATA-4, GATA-6, GATA-1 and Nkx2.5. In agreement, previous studies also observed that Art27 is frequently involved in protein interactions [12], [14], [15], [16], [17]. Art27 has high homology to the prefoldin family of molecular chaperones. Prefoldin chaperones are involved in numerous protein binding functions including protein folding and molecular signalling pathways [32], [33]. In fact, URI a member of the prefoldins, functions as a molecular scaffolding protein that assembles a multi-protein molecular complex that has functions in transcription and post-translational modification [34]. It is interesting that the cardiac cofactors of Art27 identified here are also well known to interact with one another and other crucial cardiac transcription factors [4], [25], [35], [36], [37], [38], [39], [40], [41]. Our findings suggest that it is possible Art27 participates in this interactive network, perhaps in a similar way to URI as a molecular scaffolding protein.

Our experiments identified Art27 as an intrinsic transcriptional repressor whether it was artificially tethered to the promoter by a GAL-DBD fusion or whether it was in concert with other DNA-binding cardiac transcription factors. In contrast to this, other research identified Art27 as a transcriptional activator, as a cofactor to the Androgen Receptor in prostate cancer cell lines [12]. However in these prostate cancer cells when Art27 expression was repressed, Androgen Receptor target genes were up-regulated indicating a native Art27 repressive role [20]. Art27 is likely to have a tissue specific or gene specific regulatory mechanism. This is not uncommon; other examples of this are apparent in cardiac gene regulation where Nkx2.5 generally activates genes involved in cardiomyocyte differentiation while it represses genes involved in progenitor proliferation [42]. Nevertheless, when present with cardiac transcriptional activators, Art27 is seen to dramatically down-regulate the cardiac promoters.

The transcriptional cofactors of Art27 identified in this study have roles in cardiac development [3], [8], [41], [43] and in the adult heart, regulating cardiac hypertrophic remodelling [2], [11], [44], [45]. Similarly, we have identified Art27 as a GATA-1 cofactor, which is a prominent regulator of haematopoiesis [46]. Future work is required to ascertain the biological relevance of Art27 as a transcriptional co-repressor in cardiac and other tissues. As an initial step, here we report microarray analysis of P19CL6-Mlc2v-GFP cardiomyocytes in which Art27 was knockdown by siRNA. The data show upregulation of important cardiac genes. Sfrp4 is of particular interest since it is involved, together with Wnt11, in non-canonical Wnt signalling which is in turn critical for cardiogenesis [30], [31]. Moreover, Wnt11 is a direct target of GATA4 and GATA6 and it is functionally active downstream of GATA4 and GATA6 [47], thus implicating Art27 in the regulation of endogenous GATA4 target genes.

Collectively, this work has been valuable in identifying Art27 as a novel transcriptional regulator of cardiac genes and cardiac transcription factors. This may be useful for broader application in cardiac biology research, including areas of cardiac development and cardiac hypertrophy. Research in these areas may eventually contribute to the generation of new therapies in areas of congenital heart disease, cardiac tissue engineering/regeneration and cardiac dysfunction.

## Methods

### Plasmid Constructs

The parental pCS2+ vector was kindly provided by Sergei Tevosian (Dartmouth Medical School, Hanover, NH). pCS2+FOG-2 was kindly provided by Alan Cantor (Children’s Hospital, Boston, Massachusetts). pcDNA3-Nkx2.5 was kindly provided by Mona Nemer (University of Ottawa, Canada). Human GATA-4, Art27 and Art27/GAL(DBD) were cloned into the pCS2+ expression plasmid. The BNP, ANP and αMHC luciferase reporter constructs, GATA-1 and GATA-6 were cloned into the pcDNA3.1 expression plasmid (Invitrogen). FOG-2 and Art27 GST fusions were created by subcloning into pDEST15 (Gateway Technology, Invitrogen). FOG-2, FOG-2 (856–1156), GATA-4, GATA-4 1–216, GATA-4 217–440, GATA-4 N-finger and GATA-4 C-finger were created by subcloning into pGBKT7 (Clontech). Art27 was subcloned into pACT2 (Clontech). pGBKT7-p53, pGADT7-T antigen, and pGBKT7-Lamin C were purchased from Clontech. pGEM-T:easy vector was purchased from Promega.

### Cell Culture

293a cells (Invitrogen) were grown in DMEM (Gibco) supplemented with 10% foetal calf serum (Bovogen Biologicals). Transfection of 293a was done with Lipofectamine 2000 following manufacturer’s instructions (Invitrogen). P19CL6 (with stably incorporated Mlc2v-GFP) was kindly provided by Christine Mummery (Hubrecht Laboratory, The Netherlands) and were cultured in αMEM (Gibco) supplemented with 10% foetal calf serum. P19CL6 differentiation was induced as described previously [21], [26]. Briefly, 3.7×10^5^ cells were plated into 6 cm culture dishes normal growth media +1% DMSO. Media was replenished every 48 h and extent of differentiation was tracked microscopically by GFP fluorescence, morphology and beating behaviour. GFP fluorescence was first detected at day 4 and increased up until the appearance of cardiomyocyte beating at day 10. The experiment was terminated at day 15.

### Luciferase Assay

All samples were transfected with Lipofectamine 2000 reagent (Invitrogen) as per manufacturer’s instruction. 0.1–0.5 µg of each expression vector was transfected. Each sample had equal amounts of DNA using PCS2+ backbone to equalise and 500 ng of internal control pSV-β-GAL (Promega). Cells were incubated for 40 h post transfection and 200 µl of 1x Reporter lysis buffer was added (Promega). Plates were agitated at 200 rpm for 10 minutes. 20 µl of crude cell lysates were analysed using the Dual luciferase assay promoter system (Promega) as per the manufacturer’s instructions except that renilla luciferase reading was not conducted. Normalisation of transfection efficiency was done with 50 µL of crude lysate in the Beta-Galactosidase Enzyme Assay Kit (Promega). Results presented are the average fold increase in luciferase activity (in relation to empty vector transfected samples) ± standard deviation. Data presented is representative from at least three independent experiments.

### GST Pulldown Assays

GST fusion proteins were expressed in bacteria and purified from lysates by co-incubation with GST-sepharose beads (Amersham). All purified proteins were validated by SDS-PAGE. GST pulldown assays were performed by mixing GST-sepharose bound proteins with *in vitro* ^35^S-labeled protein (TNT T7 Quick Coupled Transcription/Translation System (Promega) in GST-pulldown buffer (150 mM NaCl, 20 mM Tris-HCl, 0.1% NP-40, 2.5 mg/ml BSA, 10 nM ZnSO4, 1 mM PMSF, 0.01% β-Mercaptoethanol). Bead suspensions were resolved by 10% SDS-PAGE and exposed to phosphorimaging screens. The screen was developed on a Personal FX phosphor-imager (BIORAD) and analysed using Quantity One software (BIORAD).

### Yeast-2-hybrid

The yeast-two hybrid assay was performed using the Matchmaker GAL-4 Two-Hybrid System 3 (Clontech) as per the company instructions. Physical interactions were determined by reporter activation on SD/-Adenine/-Histidine/-leucine/-tryptophan/X-α-GAL plates. This minimal media is the high stringency test and identifies strong physical reactions. The FOG-2 C-term bait construct was used to screen a human heart library of 3.5×10^6^ independent clones. This human heart library is a representation of the genes expressed in the adult heart from generated from 3 male Caucasian donors aged 28 to 47 years. The yeast transformations for this library screen were done sequentially to ensure the highest possible efficiency, with the bait transformed into yeast alone, transformants selected, and subsequent transformation of the library. Positive interactions were rescued from yeast and PCR amplified for DNA sequencing and identification.

### Northern Blotting

∼300 bp oligonucleotide cDNA Probes were radiolabelled with 20 µCi of [a^32^P]-dCTP using a nick-translation kit (Amersham) as per the manufacturer’s instructions. RNA used in northern blot experiments was harvested with Trizol reagent (Invitrogen). 15 µg RNA was subject to electrophoresis in a 1% agarose/formaldehyde/ethidium bromide gel in 1×MOPS buffer (Sigma) and transferred to Hybond N^+^ nylon membrane (Amersham).

### Real-time PCR

RNA was isolated using the RNeasy Protect Cell Mini Kit (Qiagen). Reverse transcription of cDNA templates from total RNA was achieved using the Superscript III First-Strand Synthesis Supermix for qRT-PCR (Invitrogen). Real time PCR was conducted using Platinum SYBR Green qPCR SuperMix UDG (Invitrogen). Primers used are as follows: Gapdh; CTGAGTATGTCGTGGAGTCT, GTGGATGCAGGGATGATGTT, GATA-4; GAAGACACCCCAATCTCGAT, GCATTACATACAGGCTCACC, FOG-2; CCTTTCCTGTCTCAGTTTGC, CAGGATGTTTGAAGGAGTGG, Art27 CAAGGTATATGAGCAGCTGG, ATGCGTGAAGTATCTGGGAC, NKX2.5; GGATAAAAAAGAGCTGTGCG, TGCGTGGACGTGAGCTTCAG. All reactions were run on the Rotor-Gene 3000 Thermal cycler (Corbett Research) and analysed using REST 2009 software (Qiagen) on RG mode for relative quantitation analysis.

### siRNA Silencing, Quantitative Real Time PCR and Micro-array Assay

P19CL6-Mlc2v-GFP cells were induced to undergo cardiomyocyte differentiation with 1% DMSO as described previously [22]. Differentiation was assessed by eGFP expression with a BD FACSCanto II flow cytometer (BD Biosciences). On Day 7 or 8 of differentiation cells were harvested and used for siRNA nucleofection.

5×10^5^ Day 7 or Day 8 P19CL6-Mlc2v-GFP cells were nucleofected with 20 pmol of either control siRNA (Mission eGFP esiRNA, EHUEGFP, Sigma-Aldrich) or 20 pmol of test siRNA (Mission mouse UXT (ART27) esiRNA EMU019401, Sigma-Aldrich), with a 4D-nucleofector System (Lonza, Basel, Switzerland) using solution SG, program DS-137. After nucleofection cells were cultured in alpha MEM containing 1% DMSO.

36 h after nucleofection, total RNA was purified using the RNeasy Plus Mini Kit (Qiagen), and first-strand cDNA was synthesized from 250 ng of total RNA with Superscript III RT and oligonucleotide dT primer. All experiments were done with three biological replicates.

Quantitative real time PCR (qPCR) was performed using the StepOnePlus™ real-time PCR system (Applied Biosystems, Courtaboeuf, France) and Platinum SYBR Green qPCR SuperMix UDG (Invitrogen). Primers used for measurement of target mRNA expression are as follows: GFP-Forward, TGACCCTGAAGTTCATCTGC; GFP-Reverse, GAAGTCGTGCTGCTTCATGT; mArt27F, TTTGGGCTGTAACTTCTTCGT and mArt27R, ATATTCATGGAGTCCTTGGTG.

Nucleic acid labelling protocol: We used NuGen’s Ovation Pico WTA System V2 (NuGen, CA). Labelling, hybridisation to Affymetrix Mouse Gene 2.0 Arrays was performed by the Ramaciotti Centre for Genomics (University of New South Wales, Sydney, Australia). Briefly, 5 ng of total RNA was amplified using NuGen’s Ovation Pico WTA System V2 fragmented and labelled using NuGen’s Encore Biotin Module. 5 µg to fragmented, biotin labelled ssDNA was hybridised for 16 h at 45° at 60 rpm using an Affymetrix hybridization oven to an Affymetrix Mouse Gene Array 2.0ST (Affymetrics, CA). The arrays were washed according to manufacturers instructions on an Affymetrix FS450 fluidics station and scanned on an Affymetrix Scanner GC3000 7G. Data was analysed with GenePattern software Version 3.2.3 (Broad Institute, MIT) [48]. The data were normalized using the NormalizeAffymetrixST module followed by differential gene expression analysis using the LimmaGP module (version 19.3, available at pwbc.garvan.unsw.edu.au/gp). P≤0.001 and P≤0.01 were considered very significant and significant, respectively.

Microarray data are available in the ArrayExpress database (www.ebi.ac.uk/arrayexpress) under accession number E-MTAB-2337.

### Western Blotting

For transfected 293a cells, nuclear lysates were analysed. Membranes were probed with the following primary antibodies: rabbit anti-GATA-4 (sc-9053; Santa Cruz; 1∶200), goat anti-Nkx2.5 (AF2444, RnD systems, 1∶200) or mouse anti-β-actin (clone AC-15, Sigma, 1∶5000). The secondary antibodies used were anti-rabbit-HRP Ab (sc-2054; Santa Cruz; 1∶5000), anti-mouse HRP Ab (P0260; DAKO, Denmark; 1∶3000) or anti-goat HRP Ab (P016010/29A; DAKO, Denmark; 1∶2000). Signals were detected using Western Lightning Chemiluminescence Reagent Plus (Perkin Elmer, Wellesley, MA) and CL-Xposure Film (Quantum Scientific, QLD, Australia).

## Supporting Information

Figure S1 **Art27 physically interacts with the zinc fingers of GATA-4. (A)** *In vitro* translated and ^35^S radiolabeled GATA-4 deletion mutants containing amino acids 1–216 or amino acids 217–240 protein were incubated with full length Art27/GST fusion protein (GST-Art27) or GST only (GST-control) that was immobilised on glutathione sepharose beads. After extensive washing and electrophoresis phosphorimaging identified that ^S^35 labelled GATA-4 (aa 217–440) was caught by the GST-Art27 indicating that they physically interact but GATA-4 (aa 1–216) failed to interact. **(B)**. AH109 yeast were transformed with the respective bait and prey constructs and plated on synthetic dropout media lacking adenine, histidine, leucine and tryptophan and tested for X-GAL positive yeast growth. The physical interaction between p53 and T-antigen promoted yeast growth (segment 1- positive control), and as expected the T-antigen and Lamin C failed to promote growth (segment 2 negative control). Art27 and both GATA-4 N terminal zinc finger (segment 3) and C terminal zinc finger (segment 4) promote yeast growth indicating they physically interact.
(TIF)

Figure S2 **Art27 does not impair plasmid driven gene expression.** 293a cells transfected with expression plasmid as indicated were subjected to immunoblotting for transgene protein expression **(A)**. Cells transfected with GATA-4 expression plasmid have equal GATA-4 expression when Art27 is untransfected or cotransfected. **(B)**. Cells transfected with Nkx2.5 expression plasmid have equal Nkx2.5 expression when Art27 is untransfected or cotransfected. β-actin is used as a loading control.
(TIF)
