# Supplementary figures and images for: Art27 Interacts with GATA4, FOG2 and NKX2.5 and Is a Novel Co-Repressor of Cardiac Genes
Source: PLoS One. 2014 Apr 17;9(4):e95253. doi: 10.1371/journal.pone.0095253 (PMC3990687; doi:10.1371/journal.pone.0095253)

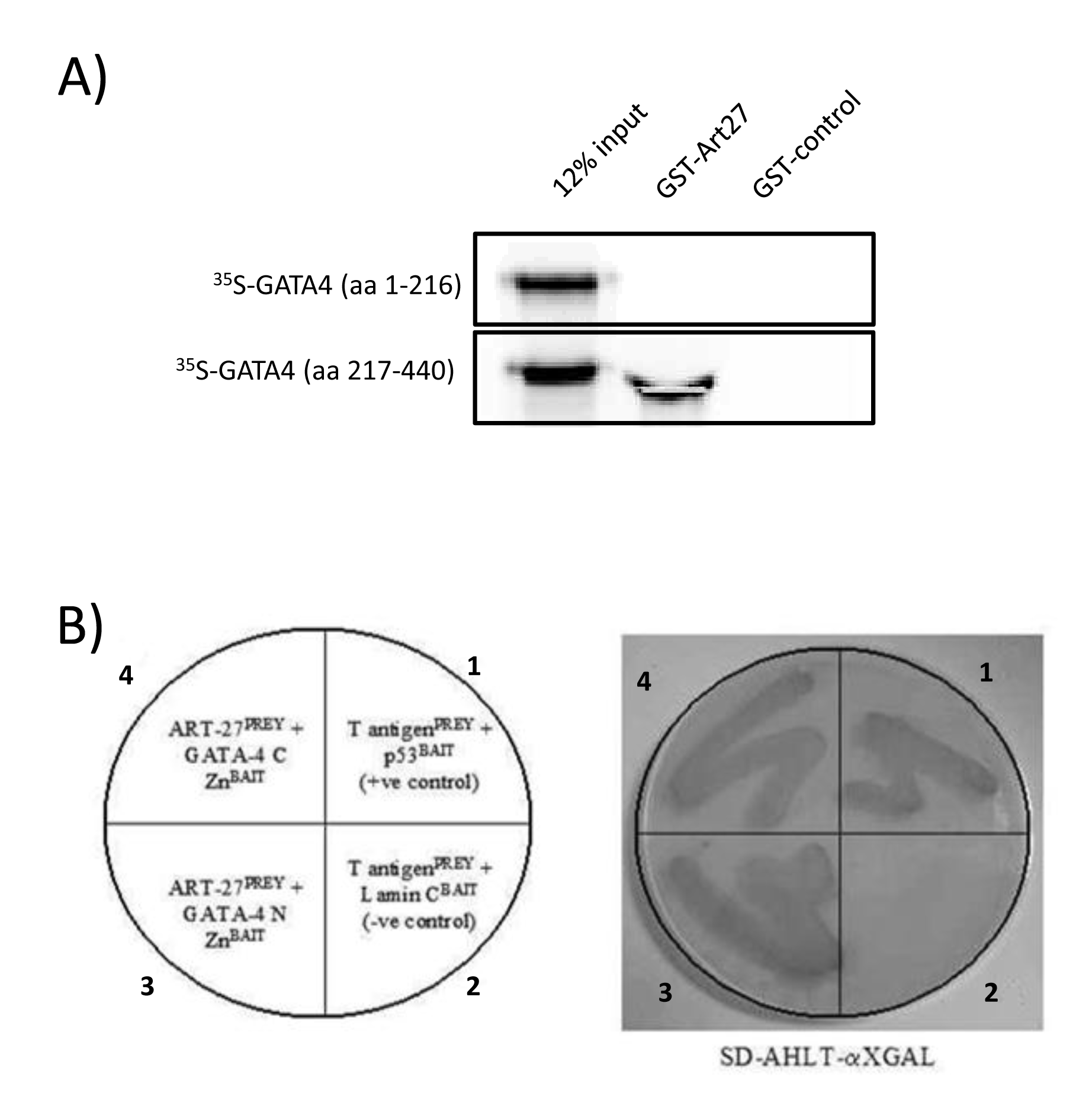

Supplement: Figure S1 — Art27 physically interacts with the zinc fingers of GATA-4. (A) In vitro translated and 35S radiolabeled GATA-4 deletion mutants containing amino acids 1–216 or amino acids 217–240 protein were incubated with full length Art27/GST fusion protein (GST-Art27) or GST only (GST-control) that was immobilised on glutathione sepharose beads. After extensive washing and electrophoresis phosphorimaging identified that S35 labelled GATA-4 (aa 217–440) was caught by the GST-Art27 indicating that they physically interact but GATA-4 (aa 1–216) failed to interact. (B). AH109 yeast were transformed with the respective bait and prey constructs and plated on synthetic dropout media lacking adenine, histidine, leucine and tryptophan and tested for X-GAL positive yeast growth. The physical interaction between p53 and T-antigen promoted yeast growth (segment 1- positive control), and as expected the T-antigen and Lamin C failed to promote growth (segment 2 negative control). Art27 and both GATA-4 N terminal zinc finger (segment 3) and C terminal zinc finger (segment 4) promote yeast growth indicating they physically interact. (TIF) [file pone.0095253.s001.tif]

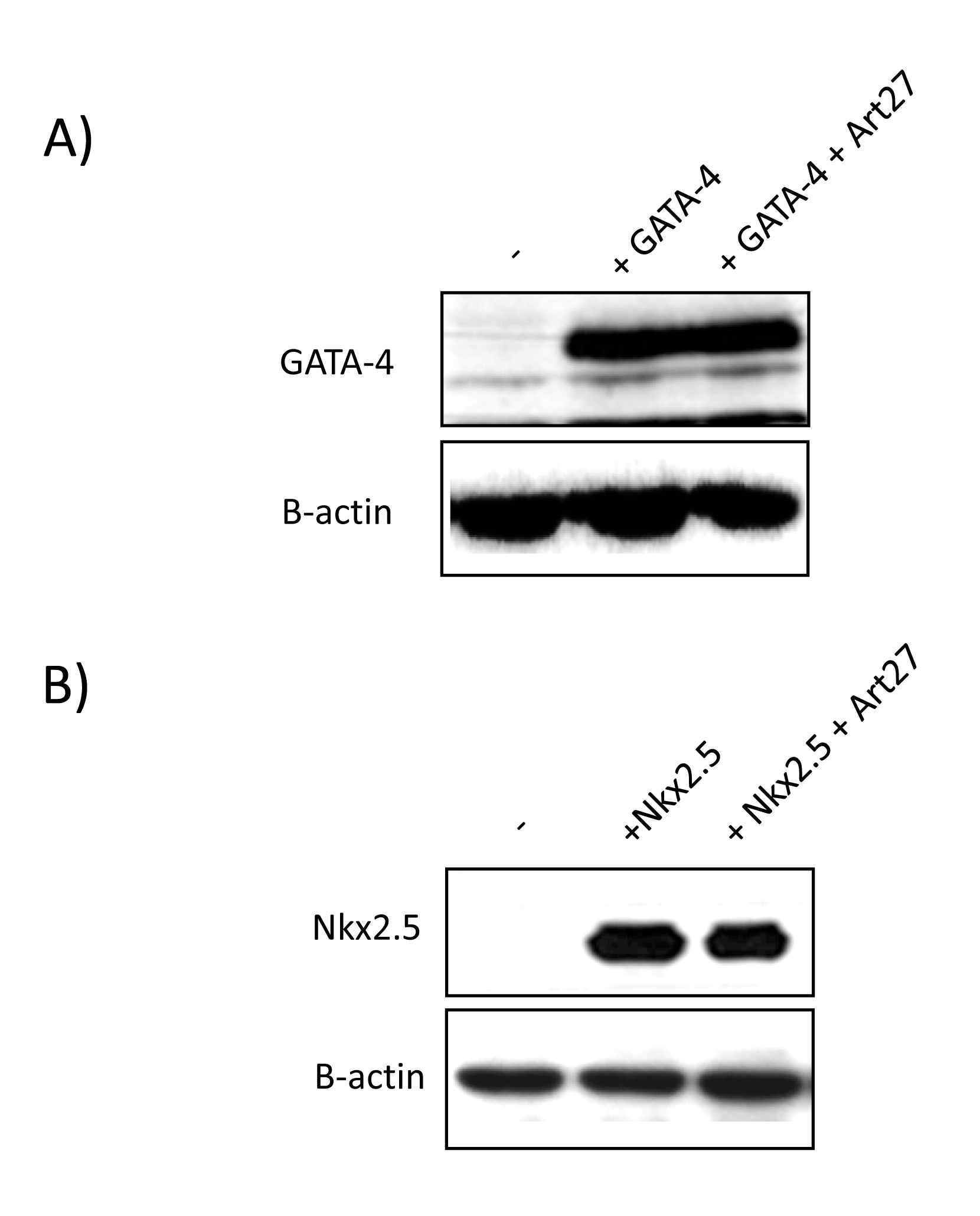

Supplement: Figure S2 — Art27 does not impair plasmid driven gene expression. 293a cells transfected with expression plasmid as indicated were subjected to immunoblotting for transgene protein expression (A). Cells transfected with GATA-4 expression plasmid have equal GATA-4 expression when Art27 is untransfected or cotransfected. (B). Cells transfected with Nkx2.5 expression plasmid have equal Nkx2.5 expression when Art27 is untransfected or cotransfected. β-actin is used as a loading control. (TIF) [file pone.0095253.s002.tif]
